# Reemergence of Sylvatic Dengue Virus Serotype 2 in Kedougou, Senegal, 2020

**DOI:** 10.3201/eid3004.231301

**Published:** 2024-04

**Authors:** Idrissa Dieng, Maryam Diarra, Bacary Djilocalisse Sadio, Bocar Sow, Alioune Gaye, Amadou Diallo, Martin Faye, Marie Henriette Dior Ndione, Diawo Diallo, Safietou Sankhe, Mignane Ndiaye, Fode Danfakha, Boly Diop, Amadou Alpha Sall, Gamou Fall, Oumar Faye, Cheikh Loucoubar, Ousmane Faye, Scott C. Weaver, Mawlouth Diallo, Mamadou Aliou Barry, Moussa Moise Diagne

**Affiliations:** Institut Pasteur de Dakar, Dakar, Senegal (I. Dieng, M. Diarra, B.D. Sadio, B. Sow, A. Gaye, A. Diallo, M. Faye, M.H.D. Ndione, D. Diallo, S. Sankhe, M. Ndiaye, A.A. Sall, G. Fall, Oumar Faye, C. Loucoubar, Ousmane Faye, M. Diallo, M.A. Barry, M.M. Diagne);; Ministry of Health Kedougou Medical Region, Kedougou, Senegal (F. Danfakha);; Ministry of Health Prevention Department, Dakar (B. Diop);; University of Texas Medical Branch, Galveston, Texas, USA (S.C. Weaver)

**Keywords:** sylvatic dengue virus serotype 2, dengue, outbreak, mosquito, vector-borne infections, viruses, zoonoses, Kedougou, southeastern Senegal, Senegal

## Abstract

In 2020, a sylvatic dengue virus serotype 2 infection outbreak resulted in 59 confirmed dengue cases in Kedougou, Senegal, suggesting those strains might not require adaptation to reemerge into urban transmission cycles. Large-scale genomic surveillance and updated molecular diagnostic tools are needed to effectively prevent dengue virus infections in Senegal.

Kedougou, Senegal’s southeastern region, is a substantial arbovirus hotspot (*1*,*2*). Decades of comprehensive surveillance have existed through both a nationwide Syndromic Sentinel Surveillance Network (*3*) and passive surveillance in several public health facilities in Kedougou and Saraya districts (*2*). Whole blood samples collected from healthcare sites are routinely sent to the World Health Organization Collaborating Center for Arboviruses and Hemorrhagic Fever Viruses at Institut Pasteur de Dakar (Dakar, Senegal) for laboratory analysis of arboviruses, as previously described (*2*,*4*). We report the reemergence of sylvatic dengue virus serotype 2 (DENV-2) in Kedougou, Senegal. The study was conducted according to the guidelines of the Declaration of Helsinki and approved by the National Ethics Committee for Health Research in Senegal (protocol no. SEN20/08, approved April 6, 2020).

## The Study

A 27-year-old man with arbovirus infection syndrome was admitted to Military Camp in Kedougou, Senegal, in November 2020. We amplified dengue virus (DENV) RNA from serum samples by using a pan-DENV 1-step quantitative reverse transcription PCR (qRT-PCR) (*4*), which confirmed dengue virus infection. Arbovirus surveillance showed 36 additional dengue cases, 27 of which had qRT-PCR–positive samples. An investigation team from Senegal’s Ministry of Health and Institut Pasteur de Dakar mobilized in December 2020 and identified 14 recently infected persons out of 42 suspected cases through retrospective tracing of health center patient records. During early December 2020 through late January 2021, a total of 4 additional qRT-PCR–positive and 4 serologically confirmed dengue cases were reported through passive surveillance.

We developed a working case definition as previously described (*5*) for suspected cases (sudden onset of fever with arbovirus symptoms) and confirmed cases (infection confirmed by laboratory methods). We conducted door-to-door case research in housing areas and collected sociodemographic and clinical data to identify infected contacts and implement effective virus spread control alongside preventive entomologic measures to eliminate mosquito breeding sites. We summarized continuous variables as means or medians and dichotomous or categorical variables as percentages with 95% CIs, as previously described (*3*). We used the Kruskal-Wallis test to compare the median ages of negative and confirmed dengue case-patients. When appropriate, we used the Pearson χ^2^ or Fisher exact test to compare percentages between categories. A p value <0.05 was considered statistically significant. We performed statistical analyses by using Stata 15 software (StataCorp LLC, https://www.stata.com).

**Figure 1:**
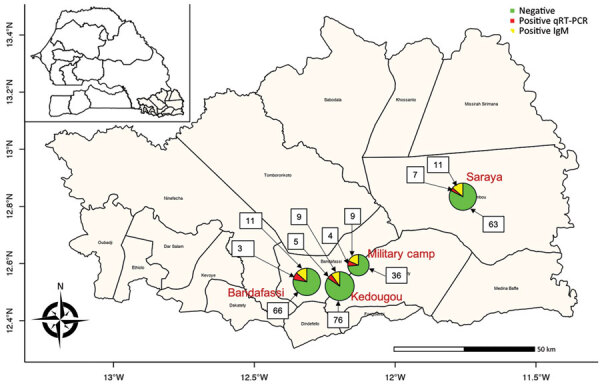
Distribution of reported cases within 4 healthcare centers during the sylvatic dengue outbreak in Kedougou region, Senegal, during November 2020–February 2021. Inset shows the Kedougou region in the southeastern corner of Senegal. Patient samples were positive according to qRT-PCR or dengue virus IgM assays of serum samples. Numbers in squares indicate the number of negative and positive cases. qRT-PCR, quantitative reverse transcription PCR.

During November 2020–February 27, 2021, we collected a total of 300 serum samples from different localities across Kedougou (Figure 1). Overall, DENV infection was found in 59 of 300 (19.6%, 95% CI 15.1%–24.2%) samples, corresponding to 32 qRT-PCR–positive and 27 IgM-positive cases. The highest number of dengue cases was recorded in Saraya health district (n = 18), followed by Bandafassi primary health center (n = 14), Kedougou health district (n = 14), and Military Camp (n = 13) (Figure 1).

Men were more affected by DENV than women; the sex ratio was 5.5:1 for confirmed cases (p = 0.005 by Pearson χ^2^ test). The mean age of all patients was 25.5 (SD +13.8) years; most (47.4%) case-patients were within the 30–45-year age group, followed by the 15–29-year (31.6%) and >45-year (1.7%) age groups. The DENV positivity rate varied significantly according to age group (p = 0.008 by Pearson χ^2^ test). Among confirmed dengue cases, the most common symptoms reported were headaches (100%; p = 0.01), followed by myalgia (57.6%) and arthralgia (47.5%) (Table 1). The sylvatic nature of the epidemic, which had potential vectors mainly outside of households, increased exposure risk for young, professionally active men working in areas at the interface of the forestry sector. Early public health measures in Kedougou comprising disinfestation campaigns have substantially reduced the number of mosquitoes in homes; however, the labor force in the region is predominantly male. In numerous countries, the number of reported incident dengue cases systematically showed a male predominance, the causes (biologic, sociodemographic, and cultural) of which deserve further investigations (*6*).

**Table 1:** Epidemiologic and clinical characteristics of suspected and confirmed dengue fever case-patients in study of reemergence of sylvatic DENV serotype 2 in Kedougou, Senegal, 2020*

Patient characteristics	Total, n = 300	DENV negative, n = 241	DENV positive, n = 59	p value
Median age, y (IQR)	25 (14.0–35.0)	24 (14.0–34.0)	29 (18.0–33.0)	0.15†
Age group, y				0.008
<15	75 (25.5)	64 (27.0)	11 (19.3)	
15–29	107 (36.4)	89 (37.6)	18 (31.6)	
30–45	88 (29.9)	61 (25.7)	27 (47.4)	
>45	24 (8.16)	23 (9.70)	1 (1.7)	
Unknown	6 (2.0)	4 (1.6)	2 (3.4)	
Sex				0.005
F	91 (30.3)	82 (34.0)	9 (15.2)	
M	209 (69.7)	159 (66.0)	50 (84.7)	
Headache				0.01
No	25 (8.3)	25 (10.4)	0 (0.0)	
Yes	275 (91.7)	216 (89.6)	59 (100.0)	
Myalgia				0.20
No	150 (50.0)	125 (52.0)	25 (42.4)	
Yes	150 (50.0)	116 (48.1)	34 (57.6)	
Arthralgia				0.85
No	161 (53.7)	130 (54.0)	31 (52.5)	
Yes	139 (46.3)	111 (46.0)	28 (47.5)	
Asthenia				0.16
No	252 (84.0)	206 (85.5)	46 (78.0)	
Yes	48 (16.0)	35 (14.5)	13 (22.0)	
Abdominal pain				0.82
No	262 (87.3)	211 (87.5)	51 (86.4)	
Yes	38 (12.7)	30 (12.4)	8 (13.6)	
Retroorbital pain				0.77
No	280 (93.3)	224 (93.0)	56 (95.0)	
Yes	20 (6.7)	17 (7.0)	3 (5.0)	
Vomiting				0.34
No	189 (63.0)	155 (64.3)	34 (57.6)	
Yes	111 (37.0)	86 (35.7)	25 (42.4)	
Investigated health facilities/regions				0.40
Kedougou health district	90 (30.0)	76 (31.5)	14 (23.7)	
Saraya health district	81 (27.0)	63 (26.1)	18 (30.5)	
Bandafassi PHC	80 (26.7)	66 (27.4)	14 (24.0)	
Military Camp	49 (16.3)	36 (15.0)	13 (22.0)	

*Values are no. (%) except as indicated. DENV, dengue virus; IQR, interquartile range; PHC, primary health center. †p value was determined by using the Kruskal-Wallis test; all other p values were determined by using χ^2^ or Fisher exact tests.

Beside human investigation, we conducted entomologic surveillance during August–November 2020 at 50 sites across 5 land cover classes (forest, barren, savanna, agricultural lands, and villages). We collected 15,937 mosquitoes, encompassing >56 species within 7 genera; >50% were known sylvatic or peridomestic DENV vectors (Table 2) (*7*). No DENV was identified in monospecific mosquito pools, whereas concomitant circulation of yellow fever virus was detected, as previously reported (*2*). Even if the same mosquitoes were screened for both viruses, larger mosquito pool sizes might be used in some tests, resulting in loss of sensitivity, which could explain the absence of DENV detection in mosquitoes during the period.

**Table 2:** Mosquito species collected during August–November 2020 in study of reemergence of sylvatic dengue virus serotype 2 in Kedougou, Senegal, 2020

Species	No. (%)
*Aedes dalzieli*	3,559 (22.3)
*Aedes furcifer*	2,332 (14.6)
*Aedes aegypti*	1,298 (8.1)
*Aedes vittatus*	971 (6.1)
*Aedes luteocephalus*	766 (4.8)
*Aedes taylori*	330 (2.1)
*Aedes africanus*	279 (1.8)
Others	6,402 (40.2)
Total	15,937 (100)

**Figure 2:**
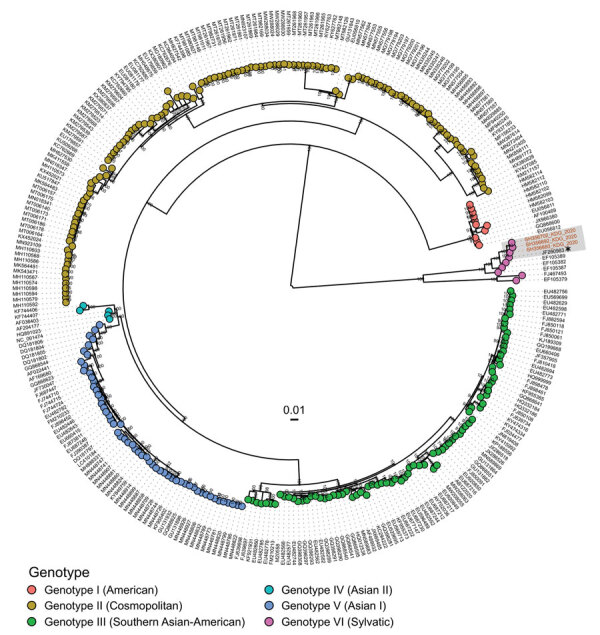
Phylogenetic analysis of dengue virus genomes in study of reemergence of sylvatic dengue virus serotype 2 in Kedougou, Senegal, 2020. Maximum-likelihood tree shows the relationships between sequenced dengue virus strains from the outbreak in Kedougou (red text) and sequences obtained from GenBank. Sequenced strains in this study belong to the sylvatic dengue virus serotype 2 genotype and are closely related to a sequence obtained in 2009 in Guinea-Bissau (asterisk).

We performed a molecular serotyping assay using specific oligonucleotide primers (Appendix Table 1) (*8*) for the pan-DENV qRT-PCR–positive human samples. We found no positive results, suggesting that the strains might belong to the DENV-2 sylvatic genotype, as previously described (*9*). We sequenced 8 samples that had PCR cycle threshold values <30 by using an amplicon-based approach on a MinION MK1C instrument (Oxford Nanopore Technologies, https://www.nanoporetech.com). We used 2 sylvatic DENV-2–specific primers pools to amplify the entire coding region of the genome. We prepared libraries by using the Rapid Barcoding Kit 96 (Oxford Nanopore Technologies) and loaded them onto an R9 flow cell. We performed data analysis as previously described (*8*). We obtained 3 high-quality sequences from 3 samples (Appendix Table 2) and aligned the consensus whole genomes with a dataset of 294 DENV-2 genotype sequences (Appendix Table 3) by using MAFFT (*10*). We built a maximum-likelihood phylogenetic tree by using IQ-TREE with default parameters and 1,000 bootstrap iterations (*11*). Phylogenetic analysis confirmed that sequenced strains belonged to the sylvatic DENV-2 genotype and were closely related to a strain identified from a traveler returning from Guinea-Bissau in 2009 (*12*) (Figure 2). In 2021, a sylvatic DENV-2 infection was reported in Kolda in southern Senegal, which is near the border with Guinea-Bissau (*9*).

## Conclusions

Although DENV in Senegal has multiple serotypes (*13*), we show that sylvatic strains are still circulating and can cause large outbreaks. Our results support previous research suggesting that sylvatic strains infecting humans might not require additional virus adaptation (*14*) but could reemerge in urban transmission cycles. Those strains should be considered as agents with epidemic potential, especially in areas such as Kedougou, where the ecosystem combines humans, nonhuman primates, and primatophilic mosquitoes (*7*,*15*). Large-scale genomic surveillance is needed, and molecular diagnostic tools should be updated for effective diagnosis and prevention of DENV infections.

Appendix Additional information for reemergence of sylvatic dengue virus serotype 2 in Kedougou, Senegal, 2020.
