# Supplementary material for: Reemergence of Sylvatic Dengue Virus Serotype 2 in Kedougou, Senegal, 2020
Source: Emerg Infect Dis. 2024 Apr;30(4):770–4. doi: 10.3201/eid3004.231301 (PMC10977847; doi:10.3201/eid3004.231301)
Supplement: Appendix — Additional information for reemergence of sylvatic dengue virus serotype 2 in Kedougou, Senegal, 2020. [file 23-1301-Techapp-s1.pdf]

Article DOI: https://doi.org/10.3201/eid3004.231301

EID cannot ensure accessibility for supplementary materials supplied by authors. Readers who have difficulty accessing supplementary content should contact the authors for assistance.

Reemergence of Sylvatic Dengue Virus Serotype 2 in Kedougou, Senegal, 2020

Appendix

Appendix Table 1. Oligonucleotide primers used in a molecular serotyping assay to retrieve sylvatic DENV-2 complete coding sequences*

Primer name	Oligonucleotide sequences, 5′–3′	GC content, %	Tm
Sylva_1_LEFT	ATGCTGAAACGCGAGAGAAACC	50.00	61.93
Sylva_1_RIGHT	GCCATTGTTGTGACGCAGCT	55.00	62.15
Sylva_2_LEFT	GTTGCTCCCTCAATGACAATGC	50.00	60.60
Sylva_2_RIGHT	ACATCCCTTTGAGTTGCAGCTT	45.45	60.94
Sylva_3_LEFT	ACATTCAAAAATCCCCATGCGAA	39.13	59.99
Sylva_3_RIGHT	GAAGCCAACTTTGAAGGGGAGT	50.00	60.94
Sylva_4_LEFT	TGATGGTGCAAGCGGACAGT	55.00	62.41
Sylva_4_RIGHT	TTTCCTGAGGCAGTGGTTGTTC	50.00	61.19
Sylva_5_LEFT	TGAAATGATAATACCAAAGAACCTGGC	37.04	60.64
Sylva_5_RIGHT	TTGTGAGGGCTAACGGTATCCA	50.00	61.34
Sylva_6_LEFT	AGTGAGGAGCATGGAAAAATACCA	41.67	60.59
Sylva_6_RIGHT	TCCACTCTCCTTCCAGTTTCCA	50.00	60.88
Sylva_7_LEFT	CATTTCACACGATGTGGCACG	52.38	60.82
Sylva_7_RIGHT	TCCATTCATGTCCGGAATTCCA	45.45	59.94
Sylva_8_LEFT	TTATGACAGCCACTCCTCCAGG	54.55	61.67
Sylva_8_RIGHT	GGATCTGAATAAGTTCTTGCGTCC	45.83	60.04
Sylva_9_LEFT	AGGAAGTGGTGCTTTGATGGAA	45.45	60.34
Sylva_9_RIGHT	GTCCATTTTTGATAGTGGCCAACC	45.83	60.94
Sylva_10_LEFT	CTGAGGCACAGCATAGAAAACTC	47.83	59.88
Sylva_10_RIGHT	CAGGCCTTTGACTTCTCTCACA	50.00	60.41
Sylva_11_LEFT	TTTCACGGGGCTCTGCAAAG	55.00	61.19
Sylva_11_RIGHT	GAAAACACGCTGCTGTCCAAAG	50.00	61.28
Sylva_12_LEFT	CCACGGCAGTTATGAGACGAAA	50.00	61.11
Sylva_12_RIGHT	TGCCCACTGCCTCTTTGATC	55.00	60.34
Sylva_13_LEFT	TATGCAGATGACACCGCTGG	55.00	60.20
Sylva_13_RIGHT	CCTAGGTATGGTATGTCTTCCCATG	48.00	60.43

*Assay used DNA from panDENV qRT-PCR–positive human samples. DENV-2, dengue virus serotype 2; qRT-PCR, quantitative reverse transcription PCR; Tm, melting temperature.

Appendix Table 2. Information and sequencing statistics for sylvatic dengue virus serotype 2 sequences generated in this study

Sample no.	Location	Sampling date	No. reads	No. mapped reads	Mean coverage depth	Genome coverage, %	Genbank accession no.
SH356683	Kedougou	2020 Nov 7	183,000	68,490	2,406.9	94.4	PP029070
SH356692	Kedougou	2020 Nov 7	143,000	84,220	3,435.9	93.9	PP029068
SH356702	Kedougou	2020 Nov 5	144,000	70,756	2,572.3	93.9	PP029069

Appendix Table 3. Dengue virus serotype 2 sequences used for phylogenetic genotyping analysis

GenBank accession no.	Genotype	Provenance
MN577555	Genotype II (Cosmopolitan)	Genbank
MN577553	Genotype II (Cosmopolitan)	Genbank
MN577556	Genotype II (Cosmopolitan)	Genbank
MG779194	Genotype II (Cosmopolitan)	Genbank
MG779198	Genotype II (Cosmopolitan)	Genbank
MG779197	Genotype II (Cosmopolitan)	Genbank
MG779203	Genotype II (Cosmopolitan)	Genbank
MG779200	Genotype II (Cosmopolitan)	Genbank
MG779196	Genotype II (Cosmopolitan)	Genbank
MG779201	Genotype II (Cosmopolitan)	Genbank
MN335247	Genotype II (Cosmopolitan)	Genbank
MN335245	Genotype II (Cosmopolitan)	Genbank
MN335244	Genotype II (Cosmopolitan)	Genbank
MN335246	Genotype II (Cosmopolitan)	Genbank
MG779195	Genotype II (Cosmopolitan)	Genbank
MG779199	Genotype II (Cosmopolitan)	Genbank
MG779202	Genotype II (Cosmopolitan)	Genbank
MN577554	Genotype II (Cosmopolitan)	Genbank
MH456892	Genotype II (Cosmopolitan)	Genbank
MH456893	Genotype II (Cosmopolitan)	Genbank
MH456895	Genotype II (Cosmopolitan)	Genbank
MH456897	Genotype II (Cosmopolitan)	Genbank
MH456898	Genotype II (Cosmopolitan)	Genbank
MN577557	Genotype II (Cosmopolitan)	Genbank
MN577560	Genotype II (Cosmopolitan)	Genbank
MN577561	Genotype II (Cosmopolitan)	Genbank
MK629885	Genotype II (Cosmopolitan)	Genbank
KM217157	Genotype II (Cosmopolitan)	Genbank
KX380828	Genotype II (Cosmopolitan)	Genbank
MW387614	Genotype II (Cosmopolitan)	Genbank
MF156233	Genotype II (Cosmopolitan)	Genbank
KY937185	Genotype II (Cosmopolitan)	Genbank
MF940245	Genotype II (Cosmopolitan)	Genbank
MF940250	Genotype II (Cosmopolitan)	Genbank
MK858111	Genotype II (Cosmopolitan)	Genbank
MN272405	Genotype II (Cosmopolitan)	Genbank
MN272404	Genotype II (Cosmopolitan)	Genbank
MH891772	Genotype II (Cosmopolitan)	Genbank
KY427085	Genotype II (Cosmopolitan)	Genbank
MT006140	Genotype II (Cosmopolitan)	Genbank
MT006173	Genotype II (Cosmopolitan)	Genbank
MT006171	Genotype II (Cosmopolitan)	Genbank
MT006178	Genotype II (Cosmopolitan)	Genbank
MT006164	Genotype II (Cosmopolitan)	Genbank
MT006186	Genotype II (Cosmopolitan)	Genbank
MT006175	Genotype II (Cosmopolitan)	Genbank
MN018341	Genotype II (Cosmopolitan)	Genbank
MT006157	Genotype II (Cosmopolitan)	Genbank
MK564483	Genotype II (Cosmopolitan)	Genbank
MN923109	Genotype II (Cosmopolitan)	Genbank
MH110568	Genotype II (Cosmopolitan)	Genbank
MH110586	Genotype II (Cosmopolitan)	Genbank
MH110603	Genotype II (Cosmopolitan)	Genbank
MH110567	Genotype II (Cosmopolitan)	Genbank
MH110574	Genotype II (Cosmopolitan)	Genbank
MH110579	Genotype II (Cosmopolitan)	Genbank
MH110582	Genotype II (Cosmopolitan)	Genbank
MH110594	Genotype II (Cosmopolitan)	Genbank
MH110598	Genotype II (Cosmopolitan)	Genbank
MK564481	Genotype II (Cosmopolitan)	Genbank
MK543471	Genotype II (Cosmopolitan)	Genbank
KX452024	Genotype II (Cosmopolitan)	Genbank
KU517847	Genotype II (Cosmopolitan)	Genbank
KX452021	Genotype II (Cosmopolitan)	Genbank
MH110573	Genotype II (Cosmopolitan)	Genbank
MN018347	Genotype II (Cosmopolitan)	Genbank
MK411558	Genotype II (Cosmopolitan)	Genbank
MH827530	Genotype II (Cosmopolitan)	Genbank

GenBank accession no.	Genotype	Provenance
KU509268	Genotype II (Cosmopolitan)	Genbank
KC762669	Genotype II (Cosmopolitan)	Genbank
EU179857	Genotype II (Cosmopolitan)	Genbank
KY794785	Genotype II (Cosmopolitan)	Genbank
KX380837	Genotype II (Cosmopolitan)	Genbank
KM279571	Genotype II (Cosmopolitan)	Genbank
KM279598	Genotype II (Cosmopolitan)	Genbank
KM279597	Genotype II (Cosmopolitan)	Genbank
KM279514	Genotype II (Cosmopolitan)	Genbank
KM279523	Genotype II (Cosmopolitan)	Genbank
KM279587	Genotype II (Cosmopolitan)	Genbank
KM279591	Genotype II (Cosmopolitan)	Genbank
KM279539	Genotype II (Cosmopolitan)	Genbank
KM279543	Genotype II (Cosmopolitan)	Genbank
KM279568	Genotype II (Cosmopolitan)	Genbank
KM279604	Genotype II (Cosmopolitan)	Genbank
KC762660	Genotype II (Cosmopolitan)	Genbank
EU081177	Genotype II (Cosmopolitan)	Genbank
EU081179	Genotype II (Cosmopolitan)	Genbank
EU081180	Genotype II (Cosmopolitan)	Genbank
KX380807	Genotype II (Cosmopolitan)	Genbank
MH048675	Genotype II (Cosmopolitan)	Genbank
KC762680	Genotype II (Cosmopolitan)	Genbank
EU179859	Genotype II (Cosmopolitan)	Genbank
MG189962	Genotype II (Cosmopolitan)	Genbank
KC762676	Genotype II (Cosmopolitan)	Genbank
KC964094	Genotype II (Cosmopolitan)	Genbank
KF744398	Genotype II (Cosmopolitan)	Genbank
MH827542	Genotype II (Cosmopolitan)	Genbank
MN577564	Genotype II (Cosmopolitan)	Genbank
MN577562	Genotype II (Cosmopolitan)	Genbank
EU056810	Genotype II (Cosmopolitan)	Genbank
MN923107	Genotype II (Cosmopolitan)	Genbank
MT261961	Genotype II (Cosmopolitan)	Genbank
MT261971	Genotype II (Cosmopolitan)	Genbank
MT982731	Genotype II (Cosmopolitan)	Genbank
MT261967	Genotype II (Cosmopolitan)	Genbank
MT980927	Genotype II (Cosmopolitan)	Genbank
MT981085	Genotype II (Cosmopolitan)	Genbank
MT981148	Genotype II (Cosmopolitan)	Genbank
MT981011	Genotype II (Cosmopolitan)	Genbank
MT261956	Genotype II (Cosmopolitan)	Genbank
MT261970	Genotype II (Cosmopolitan)	Genbank
MT261962	Genotype II (Cosmopolitan)	Genbank
MT982169	Genotype II (Cosmopolitan)	Genbank
MT261964	Genotype II (Cosmopolitan)	Genbank
MT261969	Genotype II (Cosmopolitan)	Genbank
KY627763	Genotype II (Cosmopolitan)	Genbank
MT261965	Genotype II (Cosmopolitan)	Genbank
MT261966	Genotype II (Cosmopolitan)	Genbank
KY627762	Genotype II (Cosmopolitan)	Genbank
MT261957	Genotype II (Cosmopolitan)	Genbank
MT261960	Genotype II (Cosmopolitan)	Genbank
MT261968	Genotype II (Cosmopolitan)	Genbank
MT261963	Genotype II (Cosmopolitan)	Genbank
MT261959	Genotype II (Cosmopolitan)	Genbank
MW288024	Genotype II (Cosmopolitan)	Genbank
MW288034	Genotype II (Cosmopolitan)	Genbank
MW288029	Genotype II (Cosmopolitan)	Genbank
MW288030	Genotype II (Cosmopolitan)	Genbank
MT982126	Genotype II (Cosmopolitan)	Genbank
MT982148	Genotype II (Cosmopolitan)	Genbank
GU131843	Genotype II (Cosmopolitan)	Genbank
JF260983	Genotype VI (Sylvatic)	Genbank
SH356683_KDG_2020	Genotype VI (Sylvatic)	This study
SH356692_KDG_2020	Genotype VI (Sylvatic)	This study
SH356702_KDG_2020	Genotype VI (Sylvatic)	This study
EF105389	Genotype VI (Sylvatic)	Genbank
EF105382	Genotype VI (Sylvatic)	Genbank

GenBank accession no.	Genotype	Provenance
EF105387	Genotype VI (Sylvatic)	Genbank
EF105379	Genotype VI (Sylvatic)	Genbank
FJ467493	Genotype VI (Sylvatic)	Genbank
HQ012538	Genotype III (Southern Asian-American)	Genbank
MN589883	Genotype III (Southern Asian-American)	Genbank
AF489932	Genotype III (Southern Asian-American)	Genbank
JX669485	Genotype III (Southern Asian-American)	Genbank
KC294222	Genotype III (Southern Asian-American)	Genbank
GQ868595	Genotype III (Southern Asian-American)	Genbank
GQ868541	Genotype III (Southern Asian-American)	Genbank
GQ868540	Genotype III (Southern Asian-American)	Genbank
GQ398281	Genotype III (Southern Asian-American)	Genbank
EU529695	Genotype III (Southern Asian-American)	Genbank
EU569713	Genotype III (Southern Asian-American)	Genbank
KF955373	Genotype III (Southern Asian-American)	Genbank
EU482552	Genotype III (Southern Asian-American)	Genbank
EU687244	Genotype III (Southern Asian-American)	Genbank
EU596489	Genotype III (Southern Asian-American)	Genbank
EU677147	Genotype III (Southern Asian-American)	Genbank
EU687230	Genotype III (Southern Asian-American)	Genbank
EU687222	Genotype III (Southern Asian-American)	Genbank
GQ398297	Genotype III (Southern Asian-American)	Genbank
GQ398293	Genotype III (Southern Asian-American)	Genbank
GQ398308	Genotype III (Southern Asian-American)	Genbank
GQ398291	Genotype III (Southern Asian-American)	Genbank
GQ398290	Genotype III (Southern Asian-American)	Genbank
GQ398289	Genotype III (Southern Asian-American)	Genbank
EU482562	Genotype III (Southern Asian-American)	Genbank
EU482582	Genotype III (Southern Asian-American)	Genbank
EU482744	Genotype III (Southern Asian-American)	Genbank
EU482568	Genotype III (Southern Asian-American)	Genbank
EU482577	Genotype III (Southern Asian-American)	Genbank
KY474334	Genotype III (Southern Asian-American)	Genbank
KY474316	Genotype III (Southern Asian-American)	Genbank
FJ024477	Genotype III (Southern Asian-American)	Genbank
JN819408	Genotype III (Southern Asian-American)	Genbank
GQ868641	Genotype III (Southern Asian-American)	Genbank
KF955385	Genotype III (Southern Asian-American)	Genbank
HQ332184	Genotype III (Southern Asian-American)	Genbank
FJ850108	Genotype III (Southern Asian-American)	Genbank
HQ332186	Genotype III (Southern Asian-American)	Genbank
FJ639734	Genotype III (Southern Asian-American)	Genbank
KJ189309	Genotype III (Southern Asian-American)	Genbank
FJ850061	Genotype III (Southern Asian-American)	Genbank
FJ850121	Genotype III (Southern Asian-American)	Genbank
EU569699	Genotype III (Southern Asian-American)	Genbank
EU482756	Genotype III (Southern Asian-American)	Genbank
EU482629	Genotype III (Southern Asian-American)	Genbank
EU482598	Genotype III (Southern Asian-American)	Genbank
EU482771	Genotype III (Southern Asian-American)	Genbank
FJ882594	Genotype III (Southern Asian-American)	Genbank
FJ850118	Genotype III (Southern Asian-American)	Genbank
EU660406	Genotype III (Southern Asian-American)	Genbank
GQ199868	Genotype III (Southern Asian-American)	Genbank
JF357905	Genotype III (Southern Asian-American)	Genbank
FJ810418	Genotype III (Southern Asian-American)	Genbank
EU482773	Genotype III (Southern Asian-American)	Genbank
EU482694	Genotype III (Southern Asian-American)	Genbank
HQ999999	Genotype III (Southern Asian-American)	Genbank
FJ898478	Genotype III (Southern Asian-American)	Genbank
FJ898461	Genotype III (Southern Asian-American)	Genbank
MN589869	Genotype III (Southern Asian-American)	Genbank
JX286526	Genotype III (Southern Asian-American)	Genbank
JX286518	Genotype III (Southern Asian-American)	Genbank
KP188556	Genotype III (Southern Asian-American)	Genbank
KY415992	Genotype III (Southern Asian-American)	Genbank
FJ850091	Genotype III (Southern Asian-American)	Genbank
GQ868551	Genotype III (Southern Asian-American)	Genbank
GU131864	Genotype III (Southern Asian-American)	Genbank

GenBank accession no.	Genotype	Provenance
GU131882	Genotype III (Southern Asian-American)	Genbank
EU920850	Genotype III (Southern Asian-American)	Genbank
EU687216	Genotype III (Southern Asian-American)	Genbank
AB122020	Genotype III (Southern Asian-American)	Genbank
EU920849	Genotype III (Southern Asian-American)	Genbank
MW208055	Genotype III (Southern Asian-American)	Genbank
MW208063	Genotype III (Southern Asian-American)	Genbank
EU687217	Genotype III (Southern Asian-American)	Genbank
EU687212	Genotype III (Southern Asian-American)	Genbank
AY702036	Genotype III (Southern Asian-American)	Genbank
M20558	Genotype III (Southern Asian-American)	Genbank
FJ639697	Genotype III (Southern Asian-American)	Genbank
KF921931	Genotype III (Southern Asian-American)	Genbank
FJ639698	Genotype III (Southern Asian-American)	Genbank
EU482660	Genotype III (Southern Asian-American)	Genbank
EU482778	Genotype III (Southern Asian-American)	Genbank
FM210213	Genotype III (Southern Asian-American)	Genbank
EU482785	Genotype III (Southern Asian-American)	Genbank
GQ868623	Genotype V (Asian I)	Genbank
JF730047	Genotype V (Asian I)	Genbank
NC_001474	Genotype V (Asian I)	Genbank
DQ181806	Genotype V (Asian I)	Genbank
DQ181804	Genotype V (Asian I)	Genbank
DQ181805	Genotype V (Asian I)	Genbank
DQ181802	Genotype V (Asian I)	Genbank
GQ868544	Genotype V (Asian I)	Genbank
AF022441	Genotype V (Asian I)	Genbank
AF169680	Genotype V (Asian I)	Genbank
MN448927	Genotype V (Asian I)	Genbank
MN448806	Genotype V (Asian I)	Genbank
MN448926	Genotype V (Asian I)	Genbank
MN448820	Genotype V (Asian I)	Genbank
MN448786	Genotype V (Asian I)	Genbank
MN448822	Genotype V (Asian I)	Genbank
MN448799	Genotype V (Asian I)	Genbank
MN448773	Genotype V (Asian I)	Genbank
MN448781	Genotype V (Asian I)	Genbank
MN448789	Genotype V (Asian I)	Genbank
MN448832	Genotype V (Asian I)	Genbank
GU131898	Genotype V (Asian I)	Genbank
GU131932	Genotype V (Asian I)	Genbank
GQ868625	Genotype V (Asian I)	Genbank
KF955402	Genotype V (Asian I)	Genbank
MN448744	Genotype V (Asian I)	Genbank
MN448726	Genotype V (Asian I)	Genbank
MN448728	Genotype V (Asian I)	Genbank
MN448709	Genotype V (Asian I)	Genbank
MN448858	Genotype V (Asian I)	Genbank
MN448899	Genotype V (Asian I)	Genbank
MN448887	Genotype V (Asian I)	Genbank
MN448914	Genotype V (Asian I)	Genbank
MH888331	Genotype V (Asian I)	Genbank
MN448747	Genotype V (Asian I)	Genbank
MN448741	Genotype V (Asian I)	Genbank
LC410184	Genotype V (Asian I)	Genbank
MN448881	Genotype V (Asian I)	Genbank
MN448860	Genotype V (Asian I)	Genbank
MN448824	Genotype V (Asian I)	Genbank
KY849759	Genotype V (Asian I)	Genbank
MN448907	Genotype V (Asian I)	Genbank
DQ181797	Genotype V (Asian I)	Genbank
FJ744715	Genotype V (Asian I)	Genbank
FJ744724	Genotype V (Asian I)	Genbank
FJ898452	Genotype V (Asian I)	Genbank
EU482446	Genotype V (Asian I)	Genbank
EU482643	Genotype V (Asian I)	Genbank
EU687248	Genotype V (Asian I)	Genbank
FJ390387	Genotype V (Asian I)	Genbank
FJ873811	Genotype V (Asian I)	Genbank

GenBank accession no.	Genotype	Provenance
EU660415	Genotype V (Asian I)	Genbank
EU482782	Genotype V (Asian I)	Genbank
FM210233	Genotype V (Asian I)	Genbank
FJ687447	Genotype V (Asian I)	Genbank
FJ744710	Genotype V (Asian I)	Genbank
AF038403	Genotype IV (Asian II)	Genbank
AF204177	Genotype IV (Asian II)	Genbank
HQ891023	Genotype IV (Asian II)	Genbank
KF744406	Genotype IV (Asian II)	Genbank
KF744407	Genotype IV (Asian II)	Genbank
HM582099	Genotype I (American)	Genbank
HM582102	Genotype I (American)	Genbank
HM582110	Genotype I (American)	Genbank
HM582112	Genotype I (American)	Genbank
HM582114	Genotype I (American)	Genbank
HM582103	Genotype I (American)	Genbank
GQ868600	Genotype I (American)	Genbank
JX966380	Genotype I (American)	Genbank
AF100469	Genotype I (American)	Genbank
EU056811	Genotype I (American)	Genbank
EU056812	Genotype I (American)	Genbank
